# Total body mapping in the follow-up of melanocytic lesions: recommendations of the Brazilian Society of Dermatology^☆^^☆☆^

**DOI:** 10.1016/j.abd.2020.10.005

**Published:** 2021-05-20

**Authors:** Carlos Barcaui, Renato Marchiori Bakos, Francisco Macedo Paschoal, Flávia Vasques Bittencourt, Bianca Costa Soares de Sá, Hélio Amante Miot

**Affiliations:** a Medical Sciences College, Universidade do Estado do Rio de Janeiro, Rio de Janeiro, RJ, Brazil; b Universidade Federal do Rio Grande do Sul, Porto Alegre, RS, Brazil; c Faculdade de Medicina do ABC, Santo André, SP, Brazil; d Faculty of Medicine, Universidade Federal de Minas Gerais, Belo Horizonte, MG, Brazil; e Núcleo de Câncer de Pele, A.C. Camargo Cancer Center, São Paulo, SP, Brazil; f Dermatology Discipline, Faculty of Medicine, Universidade Estadual Paulista, Botucatu, SP, Brazil

**Keywords:** Dermoscopy, Diagnostic techniques and procedures, Early cancer detection, Melanocytic nevus, Melanoma

## Abstract

Total body mapping comprises photographic documentation of the entire body surface followed by digital dermatoscopy of selected melanocytic lesions, aiming to compare their evolution over time and identify new lesions. As this is an exam based on comparative analysis of serial dermoscopic body images, standardization of the technique for performing total body mapping is essential. Prepared by specialists from the Brazilian Society of Dermatology, using the modified Delphi method, this article provides recommendations for carrying out total body mapping in Brazil, regarding its indications, technical aspects, and the issuing of the report.

## Introduction

Dermoscopy is a non-invasive diagnostic method that, when compared to the clinical examination, increases the diagnostic accuracy of melanoma (Odds Ratio = 15.6), as long as there are adequate technical skills.^1^ Digital dermoscopy consists of capturing and storing dermoscopic images. Image acquisition can be performed using different equipment that includes a digital camera attached to a dermatoscope (e.g., camera, smartphone, tablet, or videodermatoscope). Image storage allows the creation of a database that can be used to compare lesion evolution over time, for teledermatoscopy and, more recently, for diagnosis in association with artificial intelligence.^2^

Total Body Mapping (TBM), also called digital follow-up, comprises photographic documentation of the entire body surface (total body photography) followed by digital dermoscopy of selected melanocytic lesions. It is based on the fact that benign lesions remain more stable over time, while melanoma tends to grow more steadily and asymmetrically. This method, performed in two stages, when applied to high-risk patients, allows the early detection of melanoma and reduces the number of unnecessary biopsies of benign lesions.^3^ The longer the follow-up time, the greater the chance of an early diagnosis of melanoma.^4^

As this is an exam based on the comparative analysis of serial dermoscopic body images, standardization of the technique for performing TBM is essential. Aiming to establish recommendations for TBM implementation in Brazil, the Delphi method was used, after being adapted to measure the percentage of consensus based on the experience of six specialists regarding the indications, technical aspects and issuing of the report.^5^

After the choice of the topics related to TBM, made by the specialists, the recommendation texts were created based on the discussion, review of the literature, and consensus among the experts.

## Indications of total body mapping

TBM is indicated for patients with risk factors for melanoma and those with melanocytic lesions that require clinical and dermoscopic follow-up. Table 1 shows the recommended indications, the percentage of agreement between experts and the specific comments on each indication.^6^, ^7^, ^8^, ^9^, ^10^, ^11^

**Table 1:** Indications for Total Body Mapping (TBM), percentage of agreement between experts and comments on the degree of risk of melanoma occurrence in each indication.

Indications	Agreement between experts (%)	Comments
>50 common melanocytic nevi	100	Although the large number (>50) of melanocytic nevi is related to a greater risk for the development of melanoma, approximately 70% of melanomas originate in healthy skin - *de novo* melanomas. Melanomas associated with melanocytic nevi are independent from the fact of whether they are atypical or common.^6^
>5 atypical nevi	100	Atypical nevi are considered risk markers and not necessarily precursor lesions of melanoma.^6^
Previous history of melanoma	100	Patients who already had melanoma are more likely to develop another primary tumor. The relative risk is >10. The second primary melanoma occurs more frequently in the first two years after the first tumor, which probably reflects a higher level of surveillance for these patients. The probability of developing a second primary tumor is greater in patients with atypical nevus syndrome and/or a family history of melanoma.^7^
Family history of melanoma	83	The factors that cause members of the same family to develop melanoma can be genetic, environmental, behavioral, or simply random. Regardless of the cause, the family history of melanoma in a first-degree relative confers a relative risk of 1.7 to 3.0.^8^
Carriers of genetic mutations and polymorphisms associated with familial melanoma	66	Germline mutations in genes with high penetrance (CDKN2A, CDK4, BAP1) pose a risk of melanoma of 60% to 90% throughout life.^9^
		Variants R and r of the MC1R gene pose a relative risk of 1.5 to 2.9.^10^
Immunosuppressed patients	66	Those with multiple melanocytic nevi can benefit from TBM.
		Patients with altered immune systems are at increased risk of skin cancer. It has been described that patients with a history of organ transplantation using immunosuppressants have a 2 to 4-fold greater risk of melanoma than the general population.^11^
Previous history of intense and intermittent sun exposure	66	Exposure to sunlight is related to a higher incidence of melanoma, especially to the intense intermittent type and during childhood and adolescence. The presence of signs of actinic damage is an indirect way of assessing prior exposure. A previous history of sunburns is related to a relative risk of 2.0 for the development of melanoma.^8^
Indication of the exam after adolescence	100	Adolescence, according to the World Health Organization, comprises the age group between 10 and 19 years old. In specific cases, such as atypical nevus syndrome, a family or previous history of melanoma and/or due to family members’ anxiety, the exam may eventually be performed before this period, since there are no contraindications.
Performing the exam in patients being followed during pregnancy	100	Due to the structural alterations that can be observed in melanocytic lesions, for hormonal reasons and due to the increase in the volume of body parts during pregnancy, TBM should be performed when indicated, preferably, in the first trimester of pregnancy. There are no contraindications for its performance in any period of pregnancy.

## Technical aspects of total body mapping

As this is a method based on the comparison of images obtained at different times, the standardization of the technical aspects for performing the exam is crucial for TBM accuracy.

Before starting TBM, a clinical examination of the patient is recommended. The mean time to perform the first examination and the subsequent ones vary from 30 to 60 minutes. The patient most frequently adopts the decubitus position for the capture of dermoscopic images. The mean number of body photographs taken during the examination varies from 20 to 40 and the mean number of lesions included in the examination ranges from 50 to 90. Body photographs must be taken in all monitoring examinations in the long-term follow-up. It is recommended that photographs of the breasts, in women, and buttocks, palms, and plants, in both sexes, be included in TBM and photographs of the genital region, only if there are lesions that are relevant for follow-up.

The standard magnification (zoom) for capturing dermoscopic images is ×20 and the interface fluid, alcohol gel, or ultrasound gel, must be used. When the short-term follow-up (3 months) is chosen, dermoscopic documentation of all lesions is unnecessary, which should be performed only for individual lesions considered suspect and that motivated the reevaluation (Table 2).

**Table 2:** Main recommendations on the technical aspects for carrying out Total Body Mapping (TBM), percentage of agreement between the experts and comments.

Technical Aspects	Recommendation	Agreement between the experts (%)	Comments
Conducting the clinical exam before TBM	Yes	100	The previous clinical exam is of utmost importance for the selection of melanocytic lesions to be monitored.
Mean time to perform the first exam	30–60 min	83	In the first TBM, the lesions to be monitored are identified, which requires more time than the subsequent examinations.
Mean time to perform the subsequent examinations	30–60 min	100	Subsequent examinations tend to be faster because the lesions to be monitored have already been previously selected. However, more time is needed to compare the images with the previous examinations.
Patient position for the capture of dermoscopic images	Decubitus position	83	The capture of dermoscopic images can be performed with the patient in any position and does not change the accuracy of the method.
Mean number of body photographs per exam	20–40	100	Overall, the images are captured with the patient in the orthostatic posture, in the frontal, dorsal and lateral positions on both sides. Long hair should be tied up and the use of adornments should be avoided.
Mean number of lesions included per exam	50–90	100	This amount is higher than that reported by Salerni G et al. in a meta-analysis, whose average was 12 lesions, ranging from 1 to 35.^3^
The capture of total body photography in all monitoring exams	Yes	83	By comparing body photographs, it is possible to detect new lesions with or without the aid of software. The standardized image framing is very important for this evaluation.
Standard magnification (zoom) for capturing dermoscopic images	20×	100	This is the standard magnification in the most used software in Brazil and it allows performing measurements and the comparison of these data. Higher magnifications are desirable when observing or documenting specific structures. It is important that the images to be compared have the same magnification to avoid distortions.
Use of interface fluid to capture dermoscopic images	Yes	100	Alcohol gel or ultrasound gel can be used. The need to use the interface fluid also depends on the equipment used.
Only suspicious lesions should be documented in the monitoring exam during short-term follow-up.	Yes	100	This is the recommended follow-up only for individual lesions, aiming to detect subtle alterations in a short period of time (3 months). It is applied mainly for the evaluation of new lesions or those showing macroscopic alterations but without indication for excision at that moment.
Recommended time interval between exams during the long-term follow-up	6 months to 1 year	100	This interval is recommended for patients with indications for TBM who have not had suspicious lesions on examination. Follow-up is essential for the best diagnostic performance of the technique.

## Issuing of the Total Body Mapping report

TBM is considered a diagnostic imaging exam according to resolution 2235/19 of the Federal Council of Medicine, and its result must be provided as an Opinion or Report and must contain the description of the used technique, an expository section and a conclusive one.^9^ The essential data and information to be included in the TBM Report or Opinion, the percentage of agreement between the experts and the respective comments are shown in Table 3.^7^, ^8^, ^12^, ^13^, ^14^, ^15^

**Table 3:** Data and information to be included in the report or result of the total body mapping, percentage of agreement between experts and comments.

Data and Information	Agreement between experts (%)	Comments
Full name	100	As it is a medical report, the identification of the patient must be as complete as possible.
Age/Birthdate	100	It is relevant data since it can determine whether the biopsy is indicated or not according to the natural history of melanocytic nevi.
Sex	66	It is relevant data in the monitoring of melanocytic lesions, since there is variation in the incidence of melanoma in specific anatomical sites according to sex.
Previous history of melanoma	100	If positive, it indicates a higher probability of another melanoma.^7^
Family history of melanoma	100	If positive, it indicates a higher probability of the incidence of melanoma.^8^
History of genetic syndromes associated with increased incidence of tumors	66	If positive, it indicates a higher probability of the incidence of melanoma.
Description of the number of mapped lesions	100	It does not represent all of the patient's lesions, just how many were selected for follow-up.
Suggested conduct	100	According to the proposal of the International Dermatoscopy Society, it is recommended to include the conduct suggested in the report.^12^
Type of equipment used to capture images and used magnification	83	According to Art. 4 of Resolution 2235/19 of the Federal Council of Medicine, the report of diagnostic imaging exams must contain the description of the technique used.^13^
Analytical method used	83	Especially in the case of suspicious lesions, the description of the criteria that were adopted to indicate the biopsy should be stated. In Brazil, the most used analytical method is the analysis of patterns.^14^
Clinical and dermoscopic images of the lesion and description of dermoscopy	83	In addition to the images, the description of the dermoscopic findings of the individual lesions of greatest interest is recommended.
Dermoscopic nomenclature used	66	In Brazil, metaphorical terminology and not descriptive terminology is the most widely used.^15^

## Final considerations

Just like any diagnostic method, TBM has limitations. Its accuracy is directly related to the examiner’s experience. There is a consensus in the world literature that its greatest relevance is for the diagnosis of slow-growing melanomas, which present clinically as macules. It is not adequate for the follow-up of suspicious papular or nodular melanocytic lesions, although it allows the detection of their *de novo* appearance. Therefore, it is not of great help for the diagnosis of fast-growing melanoma, particularly the nodular form, which does not show the classic ABCD findings (asymmetry, irregular borders, varied color, and diameter > 6 mm), and which has high mortality rates.

As this is an exam that involves patients’ data and images, confidentiality is very important to avoid possible ethical-legal problems due to the inadequate use of images or breach of medical confidentiality.^16^

Patients with syndromes associated with an increase in the incidence of neoplasms, such as Cowden’s syndrome, Li-Fraumeni syndrome and xeroderma pigmentosum, in which the risk of melanoma is increased, may also benefit from follow-up using TBM.^17^

TBM is a medical act and, according to resolution 2235/19 of the Federal Council of Medicine, it must be carried out under the responsibility of a physician duly registered with the Regional Council of Medicine under the jurisdiction of the place where it is performed.^13^

## Financial support

Sociedade Brasileira de Dermatologia.

## Authors’ contributions

Carlos Barcaui: Idealization of work; coordination of the DELPHI group; writing, review and approval of the final version of the manuscript.

Renato Marchiori Bakos: Participation in the DELPHI group; writing, review and approval of the final version of the manuscript.

Francisco Macedo Paschoal: Participation in the DELPHI group; writing, review and approval of the final version of the manuscript.

Flávia Vasques Bittencourt: Participation in the DELPHI group; writing, review and approval of the final version of the manuscript.

Bianca Costa Soares de Sá: Participation in the DELPHI group; writing, review and approval of the final version of the manuscript.

Hélio Amante Miot: Participation in the DELPHI group; writing, review and approval of the final version of the manuscript.

## Conflicts of interest

None declared.
